# Development and implementation of a comprehensive postgraduate ultrasound curriculum for residents in obstetrics and gynecology: a feasibility study

**DOI:** 10.1007/s00404-022-06554-9

**Published:** 2022-04-16

**Authors:** Florian Recker, Martina Dugar, Paul Böckenhoff, Ulrich Gembruch, Annegret Geipel

**Affiliations:** Department for Obstetrics and Prenatal Medicine, University Hospital Bonn, Venusberg Campus 1, 53127 Bonn, Germany

**Keywords:** Ultrasound education, Obstetrics, Gynecology, Curriculum development

## Abstract

**Background:**

In obstetric and gynecological practice, ultrasound is the essential diagnostic tool. Nevertheless, few clinics have standardized and structured training curricula for young obstetricians in the field of obstetric and gynecological ultrasound. Since ultrasound is best learned hands-on in small supervised groups, we developed and implemented a comprehensive ultrasound curriculum for all postgraduate residents of our department using a peer-teaching concept.

**Methods:**

We used Kern‘s six-step model of curricular development comprising (1) problem identification and general needs assessment, (2) needs assessment of the targeted learners, (3) goals and objectives, (4) educational strategies, (5) implementation, and (6) evaluation and feedback.

**Results:**

Assistant physicians in the 1st and 2nd year of training received a theoretical and practical ultrasound basic course (six modules) in addition to their obligatory clinic rotations. The six main topics were prioritized according to service relevance and included the main features according to DEGUM, EBCOG and ISUOG. The units focused on a three-level training based on the AMEE levels: theoretical knowledge, well-founded theoretical knowledge and basic practical skills under guidance and self-employment of practical skills.

**Conclusion:**

Structured and standardized sonographic training allows young gynecology and obstetrics residents to conceptually grasp and practically implement topic-related themes. Furthermore, the course concept demonstrates the high inter-rater agreement among DEGUM-certified examiners. More research is needed to analyze the learning outcomes for residents and the improvement of the patient's outcome by establishing such an ultrasound curriculum.

## Background

The advantages of ultrasound as an imaging modality are several and include: image resolution and definition of anatomy, real-time imaging that allows immediate diagnosis and that can be precisely controlled by the operator, wide availability of ultrasound equipment and the existence of multiple simple and straightforward practical techniques covering a broad range of applications [1].

Especially in obstetrics and gynecology (OBGYN) ultrasound has a long history and is the major diagnostic tool which is used in daily practice [2]. While many obstetricians and gynecologists in training attend courses addressing the finer points, relatively few are willing to attend courses on basic theoretical and practical ultrasound techniques. The failure of basic training to keep up with diagnostic and technical developments opens the door to misinterpretation, mistakes and poor reproducibility in using the equipment. At the same time, throughout the specialty ever greater reliance is placed on ultrasound diagnoses in management for both obstetrics and gynecology [3]. Thus, the key areas for improvement are lack of sufficient training, inability to request assistance from senior colleagues when needed and human error.

Especially new residents have a lack of theoretical and practical ultrasound knowledge which are highly required in their daily clinical routine in a department for obstetrics and gynecology. There are several studies which show that a lot of practical training and a high frequency of supervision is necessary to master this clinical skill [4, 5]. We therefore aimed to develop a comprehensive postgraduate curriculum for a limited number of essential skills and practical knowledge that satisfy the demands of daily routine including night shift. Thus, at the OBGYN department of the university hospital Bonn a new training curriculum was set up.

By doing so, several aspects have to be considered in the development of an ultrasound curriculum. Given the ever increasing amount of knowledge and number of skills medical one has to tackle during the selection of learning goals is warranted [6, 7]. A successful ultrasound course should not only sample a wide range of ultrasound techniques, but also teach under which circumstances ultrasound might be the appropriate imaging technique. Performing an ultrasound examination involves theoretical knowledge about the physics of ultrasound and anatomy, pattern recognition for pathologies, as well as the ability to use the device correctly to obtain meaningful images. Thus, the range of skills that can be taught and the numbers of students is limited by the number of available instructors [8–10].

## Methods

We used Kern’s six-step approach in our development for a postgraduate ultrasound curriculum in our OBGYN department [11].

### Problem identification

To determine what best to include into an ultrasound curriculum for postgraduate gynecological and obstetrical residents, we consulted several main resources:

- The German ultrasound society (Deutsche Gesellschaft für Ultraschall in der Medizin, DEGUM) which has an own ultrasound section for Gynecology and Obstetrics. In this several quality levels are described (level I-III) with the required standard sections and fetal measurements [12–14].
- The European Board and College of Obstetrics Gynecology (EBCOG) which sets the European standard for every resident [15].
- The International Society for Ultrasound in Obstetrics and Gynecology (ISUOG) which has published international recommendations for basic training in obstetric and gynecological ultrasound [16].
- The published literature to research ultrasound skills or algorithms that are crucial for every postgraduate resident in this field and which are proposed [17].

### Needs assessment of the targeted learners

The ultrasound examination techniques identified by the above described needs assessment were discussed extensively with members of the department for gynecology and obstetrics at our hospital.

### Goals and objectives

For each identified ultrasound technique, technical, physiological and pathological properties were operationalized in January 2019. Again, clinical members with DEGUM qualification level I–III of the providing specialty were heavily involved in the process.

### Educational strategies

Since the learning goals comprised theoretical knowledge as well as practical skills, different educational strategies were blended to cover all areas and aspects. We employed the techniques developed, used and evaluated by the DEGUM for ultrasound training: scripts and lectures for the theoretical knowledge and supervised hands-on training for the practical scanning. The units focused on a three-level training based on the Association for Medical Education in Europe (AMEE) levels: theoretical knowledge, basic practical skills under guidance and self-employment of practical skills [18].

### Implementation

For the implementation, a suitable slot in the daily clinical routine was chosen and all participants of the curriculum were involved. Subsequently the infrastructure necessary to sustain the organization of the ultrasound curriculum was established.

### Evaluation and feedback

The evaluation of the comprehensive curriculum and the learning curve is made up by logbook that every resident has to fulfill. The most important standard sections have to be presented there. This includes 10 cases of fetal biometry consisting of the biparietal diameter (BPD), the fronto-occipital diameter (FOD), the abdominal transversal diameter (ATD) and abdominal sagittal diameter (ASD) and the femur length (FL). Further, five cases with the measurement of the crown-rump length (CRL) and five cases with the cervical length should be submitted. In addition, five cases each with the presentation and measurement of uterus and ovaries had to be submitted.

This documentation logbook had to be submitted within 8 weeks after the ultrasound course.

## Results

### Problem identification

In the literature, there is extensive support for minimum requirements which all OBGYN residents should obtain during their training [16, 17]. It follows that all OBGYN trainees should receive, at minimum, a basic level of theoretical and skill-based education in both obstetric and gynecological ultrasound [3]. This is particularly relevant as ultrasound technology becomes more accessible and integral to the management of women’s health [19]. The real-time, dynamic nature of ultrasound lends itself well to being performed and interpreted by obstetricians/gynecologists to facilitate timely and appropriate treatment decisions. Central to the utility of ultrasound in OBGYN is education [20].

### Needs assessment and targeted learners

**Fig. 1:**
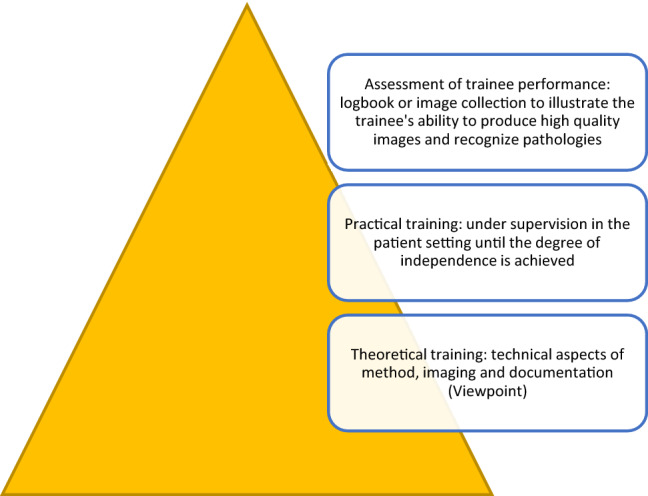
The three-step approach by establishing the comprehensive curriculum

The needed assessments were made up of a three-step approach for the residents. This approach incorporated the theoretical teaching as the fundamental basis. Further, the hands-on training was seen as the major part when focusing on night shift crucial topics. The last step was the self-made documentation (Fig. 1).

### Goals and objectives

**Fig. 2:**
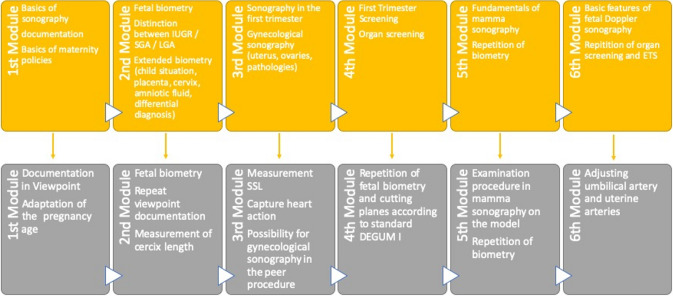
The different learning goals of the comprehensive ultrasound curriculum

The needs assessment process resulted in identifying the following goals and objectives: the successful resident will be able to (Fig. 2):

#### First module

- Describe the physical basics of ultrasound, different ultrasound probes, internal documentation software (Viewpoint GE^®^) and basics of German maternity policies.

#### Second module

- Demonstration of the major features of the fetal biometry (BPD/FOD, ASD/ATD, FL) and ultrasound presentation of these essentials.
- Use of growth charts, the distinction between intrauterine growth restricted (IUGR), small for gestational age (SGA) and large for gestational age (LGA) fetuses and to depiction of extended features, such as child presentation, normal and abnormal placentation, measurement of cervical length, assessment of amniotic fluid.

#### Third module

- Denominates the fundamental basics of the first trimester screening (measurement the crown-rump length, capturing heart beat action, non-invasive prenatal testing) and the organ screening with standard sections.
- Describes the ultrasound features of the gynecological physiology, such as standard sections and measurement of the uterus (sagittal/transversal), measurement of the endometrial thickness, presentation of the ovaries and the main pathological findings such as myomas.

#### Fourth module

- Repetition of the essential basics of the sonography in the first trimester (measurement of CRL, assessment of gestational age) and focusing on basis pathologies, such as megacystis, anencephaly, omphalocele and gastroschisis.
- Is able to perform the documentation in Viewpoint^®^ and adapts the gestational age by ultrasound.

#### Fifth module

- Depicts special applications for breast ultrasound (examination procedure) and describes fundamental sonographic features.
- Repetition of the fetal biometry and the sonographic measurement of the cervical length.

#### Sixth module

- Can adjust the umbilical artery and uterine arteries using duplex ultrasound.
- Repetition of the fetal biometry and the sonographic measurement of the cervical length.

### Educational strategies

The theoretical knowledge is conveyed in a 30–45 min lecture for all new residents performed by a senior resident or consultant at the beginning of each module. In addition, all learning goals are covered in a script that is handed to the residents. All pathologies are represented with image examples. The practical hands-on part is afterward delivered to the residents by means of a 1–2 h tutorial divided into different sessions with a ratio of one senior physician for every four to five new residents.

### Implementation

The first module was implemented in February 2019 at the OBGYN department. Involving the respective members that participated in the curriculum and in the training of the residents, a suitable slot for the implementation of the project in the curriculum was identified which was stated to be best after the normal clinical routine. Then the infrastructure to sustain the organization of the ultrasound curriculum was created. Moreover, a specific working group has been established as a long-term support for this tutor-based project. The practical share of the course formats presented was over 70 percent of the total course. The corresponding tutors were all DEGUM-certified. The tutors were present for the entire duration of the course and supported the practical training in small groups of 3–4 trainees each. The images and sectional planes created during the course were discussed directly with each participant during practice and, if necessary, the transducer guidance was also corrected individually during training. The participants practiced the sectional planes according to the DEGUM and ISOUG guidelines, which are shown in Fig. 2. The practical parts lasted approximately one-and-a-half hour per module, so that each participant had sufficient time to improve their practical ultrasound skills. The participants were all young residents who had started their careers in the past 3–6 months and who did not have basic ultrasound skills in gynecology or obstetrics.

### Feedback

**Fig. 3:**
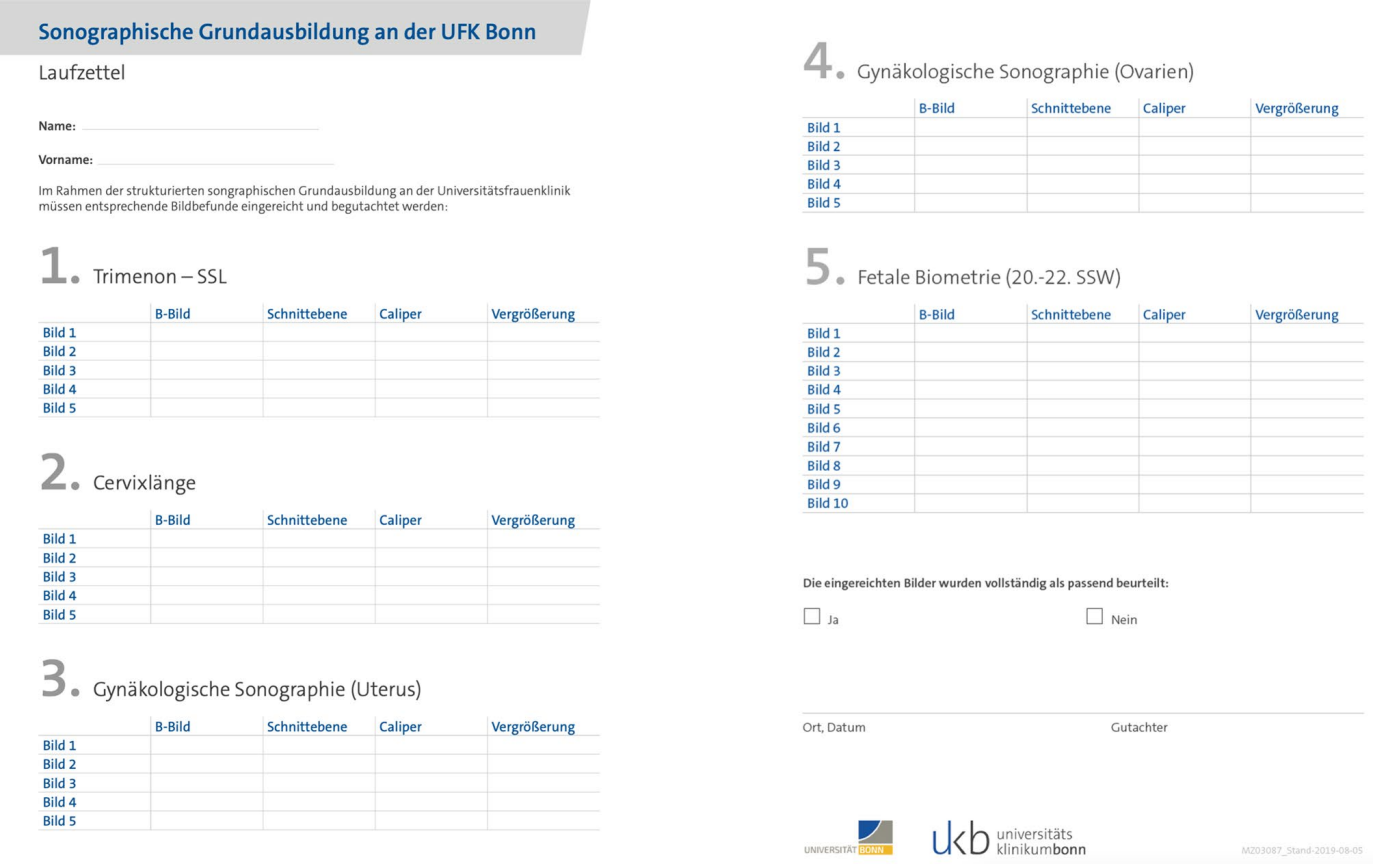
The standardized logbook for the documentation for the residents

A standardized logbook for the documentation of the most important standard sections and OBGYN ultrasound applications for the night shift was set up (Fig. 3).

14 residents took part in the training program. A completion rate with submitted pictures of 92% (13/14 participants) was achieved. The corresponding images were viewed and evaluated by DEGUM-certified examiners. Thus, a total of 390 images were rated by the examiners. Inter-reader agreement was calculated using kappa coefficients between the readers. The kappa coefficients were divided as follows: < 0.0 = poor, 0–0.20 0 slight, 0.21–0.40 = fair, 0.41–0.60 = moderate, 0.61–0.80 = substantial, and 0.81–1.0 = almost perfect agreement according to Landis and Koch [21]. The inter-reader agreement showed to be 0,8348 (95% confidence interval 0.8007–0.8688) overall. The results show that the investigators had a very good agreement when rating the images. In total, of the 390 images submitted in the course concept, the raters found 364 images to be suitable according to DEGUM standards (93.3%). This shows that the learning curve of young residents in OBGYN can be rapidly improved with the course concept shown. In addition, it demonstrates the rapid acquisition of standardized ultrasound image documentation in the field of obstetric sonography for young residents. Furthermore, the data also show that DEGUM-certified raters have a very high rate of image rating agreement, thus setting the same quality standards.

## Discussion

We used Kern’s six-step approach [11] to develop a comprehensive postgraduate ultrasound curriculum for OBGYN residents that fulfills the requirements of the DEGUM and ISUOG [16] recommendations and can be implemented and sustained for all residents.

There are several factors to be considered when implementing an ultrasound curriculum. First and foremost, knowledge about ultrasound physics, anatomy and pathologies is necessary. The scanning skills including the ability to generate an adequate image are the most crucial parts in ultrasound and are best learned by hands-on practice in very small supervised groups [8, 22].

The first task thus was to identify the most relevant skills to include in the curriculum. The skills proposed by the DEGUM and ISUOG [16] were a good starting point since they sample a large variety of techniques, for example fetal biometry, measurement of the cervical length, the use of duplex ultrasound focusing on the umbilical artery and the uterine arteries and the basic knowledge of these techniques.

A second vital consideration related to the recruitment of instructors. We decided to involve tutors from all three DEGUM qualification levels to minimize group sizes and thus maximize the hands-on time for the individual resident.

By implementing this course, we set a required standard for every new resident in our department for gynecology and obstetrics. Furthermore, we could get a harmonization of the common, in-clinic work processes by establishing this comprehensive ultrasound curriculum.

By this, we could establish a fast increasing of practical skills in the field of fetal biometry and gynecological sonography. Nevertheless, there are studies that propose between 200 and 300 supervised scans to gain a real improvement in the practical skills [23, 24]. Further, there are large differences in the learning curves for different types of examinations as some may be learned quickly whereas others require repeated practice over longer periods of time [25, 26]. These large variations underline the need for competency based education rather than ´one size fits all´ approach to ultrasound training [27]. A central concept of competency based education is to determine when trainees are sufficiently qualified for independent practice [28] which is a limitation of this comprehensive curriculum development. A weakness and limitation of the presented study is the presentation of the pure outcome of the described training format. Further studies must be conducted here to discuss the growth curve of the learning behavior with the respective pre- and post-testing. This could be done using practical ultrasound examination formats such as objective structured clinical examinations (OSCE). As a further step one could combine this developed curriculum with a published scale for assessment of ultrasound competence, the Objective Structured Assessment of Ultrasound Skills (OSAUS) [20]. This scale has shown evidence of validity and high reliability by judging the ultrasound skills competence for users.

Using a course-based training enables full control over the contents and splitting it up in a six-module-system made it adjustable to the busy timetable every new resident has at the beginning of his residency. Moreover, it makes it more adaptable to different clinical settings and is generalizable to OBGYN departments.

As described we developed a comprehensive ultrasound curriculum that at first fulfills the requirements of the DEGUM and the ISUOG, second, samples important pathologies novice physicians may encounter, and third can be implemented and sustained for all residents. Future studies now should investigate the potential impact of the curriculum on patient care especially in the night shifts.

## Data Availability

Not applicable.
